# Protein Expression Knockdown in Cancer Cells Induced by a Gemini Cationic Lipid Nanovector with Histidine-Based Polar Heads

**DOI:** 10.3390/pharmaceutics12090791

**Published:** 2020-08-21

**Authors:** Natalia Sánchez-Arribas, María Martínez-Negro, Eva M. Villar, Lourdes Pérez, José Osío Barcina, Emilio Aicart, Pablo Taboada, Andrés Guerrero-Martínez, Elena Junquera

**Affiliations:** 1 Departamento de Química Física, Facultad de Ciencias Químicas, Universidad Complutense de Madrid, 28040 Madrid, Spain; natsanch@ucm.es (N.S.-A.); mmnegro@ucm.es (M.M.-N.); aicart@ucm.es (E.A.); aguerrero@quim.ucm.es (A.G.-M.); 2 Departamento de Física de Partículas, Facultad de Físicas e Instituto de Investigaciones Sanitarias (IDIS), Universidad de Santiago de Compostela, Campus Vida, E-15782 Santiago de Compostela, Spain; eva.mailbox1@gmail.com (E.M.V.); pablo.taboada@usc.es (P.T.); 3 Departamento de Tensioactivos y Nanobiotecnología, IQAC-CSIC, 08034 Barcelona, Spain; lpmste@cid.csic.es; 4 Departamento de Química Orgánica, Facultad de Ciencias Químicas, Universidad Complutense de Madrid, 28040 Madrid, Spain; josio@ucm.es

**Keywords:** Non-viral gene delivery, gene knockdown efficiency, small interfering RNA, amino acid-based gemini cationic lipids, protein expression, protein corona

## Abstract

A histidine-based gemini cationic lipid, which had already demonstrated its efficiency as a plasmid DNA (pDNA) nanocarrier, has been used in this work to transfect a small interfering RNA (siRNA) into cancer cells. In combination with the helper lipid monoolein glycerol (MOG), the cationic lipid was used as an antiGFP-siRNA nanovector in a multidisciplinary study. Initially, a biophysical characterization by zeta potential (ζ) and agarose gel electrophoresis experiments was performed to determine the lipid effective charge and confirm siRNA compaction. The lipoplexes formed were arranged in L_α_ lamellar lyotropic liquid crystal phases with a cluster-type morphology, as cryo-transmission electron microscopy (cryo-TEM) and small-angle X-ray scattering (SAXS) studies revealed. Additionally, in vitro experiments confirmed the high gene knockdown efficiency of the lipid-based nanovehicle as detected by flow cytometry (FC) and epifluorescence microscopy, even better than that of Lipofectamine2000*, the transfecting reagent commonly used as a positive control. Cytotoxicity assays indicated that the nanovector is non-toxic to cells. Finally, using nano-liquid chromatography tandem mass spectrometry (nanoLC-MS/MS), apolipoprotein A-I and A-II followed by serum albumin were identified as the proteins with higher affinity for the surface of the lipoplexes. This fact could be beyond the remarkable silencing activity of the histidine-based lipid nanocarrier herein presented.

## 1. Introduction

The use of nucleic acids as therapeutic agents offers a wide range of possibilities with regard to the treatment of diseases at the molecular genetic level [1,2]. Specifically, the discovery of interference RNA (RNAi) and the development of siRNA molecules have allowed the possibility of controlling a specific and unique mechanism of action in the regulation of genes [3,4]. This has been translated into better results in terms of selectivity and efficiency compared to other therapeutic agents used in gene therapy, such as pDNA and oligonucleotides [5,6]. The siRNA molecules can block specific regions in the messenger RNA (mRNA) sequence through the formation of a RNA-induced silencing complex (RISC), thus suppressing the synthesis of the target pathogenic protein. However, this powerful and selective method for gene silencing can be limited or may even not take place if the siRNA molecules do not reach the cell cytoplasm. The degradation by nucleases present in the bloodstream and their inefficiency to cross the negatively charged cellular membrane are some of the limitations that make the vectorization of nucleic acids necessary.

Bioinspired non-viral vectors, which came to replace viral vectors that often suffer from limitations related to the immune response [7,8], can be functionalized with amino acids components or made of oligopeptides sequences. Cell-penetrating peptides are one of the best examples of a short peptide sequence that can pack and unpack nucleic acids into cells in a non-toxic manner [9,10]. However, efficiency achieved in in vivo experiments is not high enough provided that these peptide-based complexes possess low cell specificity [11]. Dendrimers [12,13], polymers [14,15] and lipids [16,17] are other nanocarriers among non-viral vectors that may incorporate natural amino acids in their structures to reduce cytotoxicity and improve cellular uptake. Lysine, arginine and histidine are some of the most commonly used amino acids because of their positive charge at physiological pH, which enables electrostatic interactions with the anionic nucleic acids and the negatively charged cellular membranes [16,17,18,19,20]. In particular, the histidine group also offers a mechanism for endosomal scape, known as the “proton sponge effect” [21,22,23]. This strategy has been used as a source of inspiration for histidine-based nanocarriers in the last decade [21], specifically in the case of cationic lipids (CL), which have received much attention as non-viral gene vectors thanks to their structural similarities with the cell membrane. The insertion of amino acid moieties, bare or functionalized, in their structure has been generally more used in pDNA transfection [18,20,22,24,25,26,27,28] than in siRNA vectorization [29,30,31,32], despite the fact that amphiphilic imidazolium salt has already been physico-chemically presented as a new generation of reagents for RNAi [33]. Additionally, gemini cationic lipids (GCL) have demonstrated to be efficient gene nanovectors [34,35,36], especially when an imidazolium group was included in their structure [37,38]. Both synthetic strategies are focused on overcoming the biological barriers that the nanocarrier encounters once it enters the bloodstream [39], as well as on improving the endocytosis pathway [40]. In this regard, the inclusion of a co-adjuvant lipid in the formulation is a common approach for enhancing the fusogenic properties of the complex formed by lipids and nucleic acids (lipoplex) with the cell membrane. The helper lipid habitually used in the gene-knockdown field is the 1-(cis-9-octadecenoyl)-rac-glycerol (MOG). Its biocompatibility and ability to induce different lyotropic liquid crystal phases make it a safe option in gene therapy [38,41,42,43].

However, when a nanocarrier is introduced in the body, a journey through biological fluids starts where a wide variety of molecules can interact with it, conditioning the success in achieving its target. Particularly, proteins tend to adsorb onto the nanovector surface in a dynamic process [44,45], forming a new biological entity, which is what cells firstly see. That protein corona (PC) can either trigger an immune response or favor the absorption of the nanoagent by the cell membrane [46,47,48], and, ultimately, it is thought that it largely decides the successful or, on the contrary, defective end of the gene nanocarrier. Nowadays, many studies are focused on an extensive characterization of the PC of non-viral gene nanocarriers with the aim of gaining a better understanding of its effect on the efficiency of the transfection process, which is one of the bottlenecks in in vivo treatments [49,50,51,52].

**Scheme 1:**
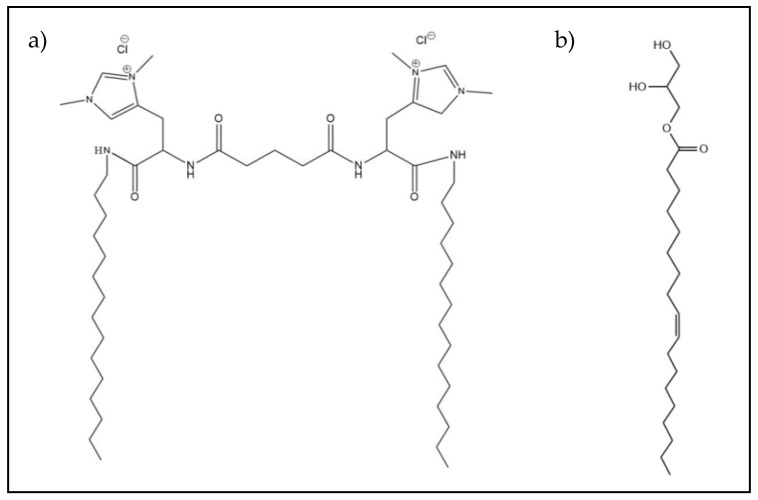
Molecular structure of the histidine-based gemini cationic lipid C_3_(C_16_His)_2_ (**a**) and the neutral lipid MOG (**b**).

Following this strategy, and taking into account previous studies of cationic lipids (CLs) incorporating amino acid derivatives in their structures such as lysine [18,53] and arginine derivatives [43], we have worked in this study with a nanovector based on a gemini cationic lipid with functionalized histidine residues on the head groups, the bis(N(τ),N(π)-bis(methyl)-histidine hexadecyl amide) propane—abbreviated as C_3_(C_16_His)_2_ (see Scheme 1a). This GCL has already been used as an interesting nanoplatform for pDNA delivery [26]. These previous promising data allowed us to hypothesize that this modified lipid can be also used to effectively transport and deliver siRNA molecules and, therefore, to enable the configuration of a versatile non-viral gene nanocarrier. Thus, a lipid mixture that contained this histidine-based GCL and the neutral lipid MOG was used to compact antiGFP-siRNA molecules. The lipoplexes formed were physico-chemically characterized by agarose gel electrophoresis, ζ potential, cryo-TEM, and SAXS techniques. The gene knockdown activity was evaluated by measuring the fluorescence signal of the green fluorescent protein (GFP) overexpressed in HeLa and T731 cancer cells through flow cytometry and epifluorescence microscopy. The cytotoxicity of lipoplexes was analyzed by the CCK-8 assay, whilst nanoLC-MS/MS experiments were performed to examine the proteomic profile of the protein corona surrounding the lipoplexes in physiological-mimicking conditions. Altogether, these in vitro experiments could confirm the potential utility of the histidine-based nanocarrier for gene knockdown therapy.

## 2. Materials and Methods 

### 2.1. Materials 

The synthesis of C_3_(C_16_His)_2_ has been previously reported [26]. The helper lipid MOG (see Scheme 1b) and Pluronic F127 (10% *w/v* in water) were supplied by Sigma-Aldrich (St. Louis, MO, USA) and ThermoFisher (Waltham, MA, USA), respectively. For cell culturing, Dulbecco’s Modified Eagle Medium (DMEM) was supplied by Hyclone-ThermoFisher (Waltham, MA, USA) while Fetal Bovine Serum (FBS), antibiotics, sodium pyruvate, and non-essential amino acids (NEAAs) were provided by Gibco-ThermoFisher (Waltham, MA, USA). Human Serum (HS) was used as received from Sigma-Aldrich. The in vitro evaluation was done using an antiGFP-siRNA and a non-targeting control siRNA (also known as scrambled siRNA, i.e., without functional activity) supplied by Ambion-ThermoFisher and Invitrogen-ThermoFisher, respectively. Finally, the commercial control Lipofectamine2000* Transfection Reagent (Lipo2000*) was also obtained from Invitrogen-ThermoFisher.

### 2.2. Preparation of Lipoplexes

Appropriate amounts of C_3_(C_16_His)_2_) and MOG (at a molar fraction with respect to C_3_ (C_16_His)_2_), α = 0.2) were mixed to obtain dry lipid films by evaporation of chloroform under high vacuum. These dry films were afterwards hydrated with HEPES (40 mM, pH = 7.4) and further homogenized by using a procedure fully detailed elsewhere [54]. Subsequently, a sequential extrusion procedure, fully detailed elsewhere [55], was used to favor a population of unilamellar liposomes with low polydispersities [56,57]. Finally, Pluronic F127 (10% in mass of GCL) was added to the lipid mixture to provide colloidal stability. A certain amount of siRNA was added to each stabilized lipid mixture to form lipoplexes with fixed compositions, and the whole mixtures were incubated at room temperature for at least 30 min to form the C_3_ (C_16_His)_2_/MOG-siRNA lipoplexes. The concentration of siRNA in the stock solution was: 0.1 mg/mL for ζ, 0.2 µg/well (0.1 mg/mL) for agarose gel electrophoresis, 50 µg/capillary (10 mg/mL) for SAXS, 0.2 mg/mL for cryo-TEM and protein corona studies, and 5 nmol/mL (antiGFP-siRNA) or 1 nmol/mL (non-targeting siRNA) for biological experiments.

### 2.3. Electrochemical study. Ζ Potential and Agarose Gel Electrophoresis

The ζ potential was determined at 25 °C through electrophoretic mobility measurements using the phase analysis light scattering technique (Zeta PALS, Brookhaven Instruments Corp., Holtsville, USA), fully detailed elsewhere [57,58]. ζ values of C_3_(C_16_His)_2_/MOG-siRNA lipoplexes were collected in a sigmoidal curve as a function of the mass ratio between the lipid mixture and the siRNA, $m_{L}{/m}_{\mathrm{siRNA}}$:

(1) $$m_{L}{/m}_{\mathrm{siRNA}}{=(m}_{\mathrm{GCL}}{+\;m}_{L^{0}}{)/m}_{\mathrm{siRNA}}$$

where $m_{L}$, $m_{\mathrm{GCL}}$, $m_{L^{0}}$, and $m_{\mathrm{siRNA}}$ are the masses of the total mixed lipid, the GCL (C_3_(C_16_His)_2_), the neutral lipid (MOG), and siRNA, respectively. Each value in the graph is the average of 50 independent measurements. 

Furthermore, the capacity of the lipid mixtures to complex and compact siRNA molecules was determined by a compaction assay with agarose gel electrophoresis. Free siRNA and C_3_(C_16_His)_2_/MOG-siRNA lipoplexes at different compositions were included in a 0.8% (*w/v*) agarose gel in 1X TAE buffer, and 0.7 µL of GelRed probe added. Electrophoresis was run at room temperature (around 25 °C) at 70 mV for 1 h. Gels were visualized using a Gel Doc XR instrument (Bio-Rad) under Quantity One software; probe emission was excited at 302–312 nm and recorded at 600 nm. The presence of free or uncompacted siRNA is detected by a characteristic fluorescent band of the probe intercalated within the siRNA double helices, while its absence denotes full siRNA compaction by the lipid mixture.

### 2.4. Structure Study. SAXS and Cryo-TEM

SAXS experiments were carried out at ALBA Synchrotron (Barcelona, Spain, beamline BL11) with an incident beam energy of 12.6 KeV (λ = 0.995 Å) and a Quantum 210r CCD detector. Diffractograms collected the scattered X-rays signal, converted into one-dimensional scattering by radial averaging, as a function of the momentum transfer vector (q). The lipoplexes were incubated and analyzed in the absence and presence of human serum, HS (10% *v/v*). Samples were measured in duplicate for each composition.

C_3_(C_16_His)_2_/MOG-siRNA lipoplexes were also deposited for cryo-TEM experiments on perforated Holey Carbon on a 400-mesh copper grid. Following a previously reported protocol [59,60,61], samples were observed using a JEOL JEM 2011 microscope at 200 kV under low-dose conditions and with different degrees of defocus (500–700 nm). The micrographs were collected with a Gatan 794 Multiscan digital camera, and the digital Micrograph software was used to analyze the CCD images.

### 2.5. In Vitro Evaluation

#### 2.5.1. Cell Culturing

Two cancer cell lines overexpressing the green fluorescent protein, GFP, were used: cancer cervical HeLa-GFP and mouse astrocytes T731-GFP, which were obtained from Cell Biolabs (San Diego, CA, USA) and kindly donated by Prof. J. A. Costoya (Univ. of Santiago, Spain) [62], respectively. Cells were cultured at standard conditions (37 °C, 5% CO_2_) in Dulbecco’s Modified Eagle Medium, DMEM, supplemented with 10% (*v/v*) FBS or HS and 1% (*v/v*) of penicillin/streptomycin, sodium pyruvate and nonessential amino acids (NEAAs).

#### 2.5.2. Cytotoxicity, Epifluorescence Microscopy and Flow Cytometry

For the in vitro evaluation of the developed lipid-based nanocarriers, cells were seeded as follows: in 96-well plates (100 µL, 1 ×10^4^ cells/well) for cytotoxicity assays; on poly-L-lysine-coated glass coverslips (76 mm × 26 mm) placed inside 6-well plates (3 mL, 1 × 10^5^ cells/well) for epifluorescence microscopy measurements; and in 12-well plates (2 mL, 4 × 10^4^ cells/well) for flow cytometry experiments, respectively. 

After 24 h of cell seeding, C_3_(C_16_His)_2_/MOG-siRNA lipoplexes were administered to cells in culture medium supplemented with 10% (*v/v*) HS instead of FBS. Then, lipoplexes formed at a concentration of 5, 40, and 100 pmol/well of antiGFP-siRNA were incubated with the cells for 48 and/or 72 h and cytotoxicity assays, flow cytometry and epifluorescence microscopy experiments performed. Lipo2000* at 0.25, 2, and 5 µL/well and non-treated GFP-overexpressing cells were used as positive and negative controls, respectively. For flow cytometry experiments, non-targeting siRNA encapsulated in lipoplexes at a concentration of 2 µL/well of Lipo2000*, and free added antiGFP-siRNA were used as additional controls.

The cytotoxicity of C_3_(C_16_His)_2_/MOG-siRNA lipoplexes were quantified by a colorimetric assay with the Cell Counting Kit-8 (CCK-8) proliferation assay. After 48 and 72 h of incubation, cells were washed with PBS and new medium without FBS or HS added containing 10% (*v/v*) of the CCK-8 reagent. After 2 h of incubation, absorbance at 450 nm was measured with an UV−vis microplate absorbance reader (Bio-Rad, model 689). For calculation of the % of cell viability, absorbance of the treated cells was normalized regarding the absorbance of non-treated ones.

For epifluorescence microscopy measurements, cells were incubated with lipoplexes for 72 h. After washing with PBS twice, cells were fixed with 200 µL of 4% (*w/v*) paraformaldehyde for 10 min. Afterwards, the cell membrane was permeabilized with 200 µL of 0.2% (*w/v*) Triton X-100. Then, the cells were washed again, stained firstly with DAPI (Invitrogen-ThermoFisher) for nuclei for 10 min; washed again, and next the cell cytoplasm stained with Alexa Fluor^TM^ 647 (Invitrogen-ThermoFisher) for 20 min following an additional washing step. Finally, the cell-covered coverslips were mounted on glass slides and visualized after storage for 24 h at -20 °C with an epifluorescence Leica DMI6000B microscope equipped with a Leica AF6000 modular system and a DFC3665FX camera (Leica Microsystems GmbH, Heidelberg, Mannheim, Germany). An oil objective of 63X, and blue channel for DAPI (λ_ex_ = 350 nm; λ_em_ = 460 nm), far red channel for Alexa Fluor^TM^ 647 (λ_ex_ = 650 nm; λ_em_ = 668 nm) and transmitted light in differential interference contrast (DIC) mode were used to capture the images. The % of GFP expression was obtained by the analysis of the fluorescence intensities using LAS X Life Science and ImageJ softwares following an established methodology previously detailed [63]. Briefly, the selection of regions of interest (ROIs) considering well-defined cells was done in several microscopic images, and the fluorescent signal was normalized and quantified regarding the signal background. 

Finally, in flow cytometry experiments, harvested cells after 48 and 72 h of incubation were washed and resuspended in 200 µL of PBS three times (1,200 rpm for 4 min). The analysis of GFP down-regulation was done in terms of the percentage of GFP cells observed (% GFP), and the average of the fluorescence intensity per cell (mean fluorescence intensity, MFI). At least 5,000 events were counted using a Guava^®^ easyCyte HT System flow cytometer and GuavaSoft™ software.

### 2.6. Protein Ccorona Studies

The proteomic profile of proteins surrounding the surface of C_3_(C_16_His)_2_/MOG-siRNA lipoplexes was analyzed through nanoLC-MS/MS. The procedure followed was fully detailed in a previous work [43]. Briefly, the lipoplexes formed were incubated for 1 h at 37 °C in the presence of HS. After dithiothreitol reduction, iodacetamide alkylation and a recombinant trypsin digestion treatment overnight at 37 °C, peptides were eluted, concentrated and desalted in C18 reverse phase chromatography columns with acetonitrile/trifluoroacetic acid (ACN/TFA). Peptides are affinity-retained on C18 chains and recovered with 50% (*v/v*) ACN and 0.1% TFA (*v/v*). The samples were dried by vacuum centrifugation (SpeedVac, Savant) and reconstituted in 20 μL of formic acid for analysis by RP-LC-ESI-MS/MS in an EASY-nLC 1000 System coupled to the Q-Exactive HF mass spectrometer through the Nano-Easy spray source. There, samples were loaded into an Acclaim PepMap 100 Trapping pre-columm, separated and eluted on a NTCC C18 resin analytical column at a constant flow rate of 250 nL/min. Data acquisition was performed with a Q-Exactive HF mass spectrophotometer using an ion spray voltage of 1.8 kV. Then, the peptide identification from raw data was carried out using Sequest search engine through the Protein Discoverer 2.2 Software (Thermo Scientific). The percolator algorithm was used to estimate FDR < 1% for proteins identified with high confidence, and only protein identification based on mass spectra related to at least two unique peptides was considered. In the quantitative analysis of the proteins, the mean value of peptide to spectrum matches (PSMs) was normalized to the protein molecular weight in kDa (MW) and expressed as the relative percentage of proteins.

## 3. Results and Discussion

### 3.1. Electrochemical Study. Ζ Potential and Agarose Gel Electrophoresis

In the present work, the GCL C_3_(C_16_His)_2_ was used in combination with the helper lipid MOG as a nanoplatform to introduce gene material into HeLa and T731 cancer cells in an efficient and safe manner. A molar fraction (α) with respect to the GCL of 0.2, which implies a larger content of the neutral helper lipid (MOG) in the mixture, was chosen since it was revealed as optimal in previous works with similar siRNA nanocarriers [38,43]. The efficiency of the siRNA nanovector relies on a siRNA packing-unpacking mechanism conducted by the lipid mixture, which is responsible for the compaction of siRNA and its subsequent delivery inside the cells. The net charge of the lipoplex and the capacity of the nanocarrier to compact the siRNA molecule are probably among the most decisive factors to favor the transfection process.

**Figure 1:**
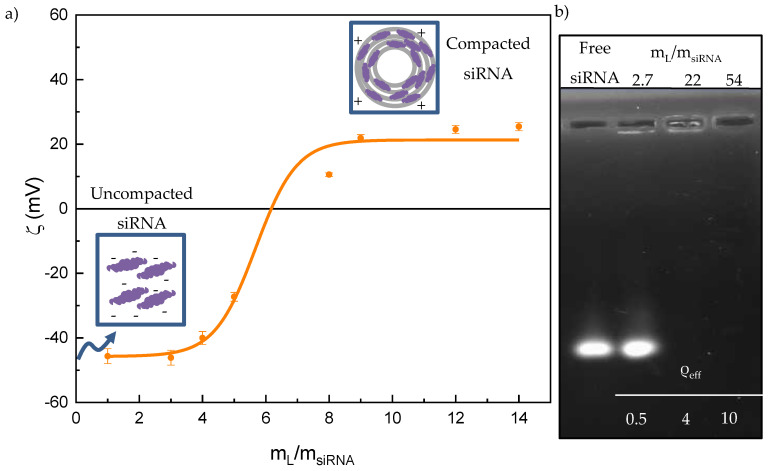
(**a**) Plot of the ζ vs. mass ratio ($m_{L}{/m}_{\mathrm{siRNA}}$) of C_3_(C_16_His)_2_/MOG-siRNA lipoplexes at α = 0.2. (**b**) Agarose gel electrophoresis of the lipoplexes formed at selected $m_{L}{/m}_{\mathrm{siRNA}}$ (or ρ_eff_) and α = 0.2 (2–4 lanes). Used as control: free siRNA (lane 1).

With respect to the electric charge, the anionic character of siRNA molecules is an important drawback to overcome since the cellular membrane is also negatively charged. The composition of the lipoplex at which its net charge changes from negative to positive, the so-called electroneutrality value, is a key information, since it marks the lower limit from which a lipoplex is potentially a suitable vector, strictly speaking from the point of view of the charge. Probably, the physico-chemical properties that allow us to obtain the electroneutrality value more accurately are either the electrophoretic mobility or the ζ potential, measured as a function of the $m_{L}{/m}_{\mathrm{siRNA}}$ ratio (at constant siRNA concentration), as reported in Figure 1a for C_3_(C_16_His)_2_/MOG-siRNA lipoplexes. These plots usually show a sigmoidal profile with three zones (see schemes included in Figure 1a): (i) the zone of net negative charge, where there is an excess of anionic siRNA molecules; (ii) the electroneutrality region that contains the electroneutrality ratio, i.e., the $m_{L}{/m}_{\mathrm{siRNA}}$ value in which lipoplexes have a net ζ (and, accordingly, the surface charge) equal to zero; and (iii) the zone of net positive charge, where there are cationic lipoplexes with the siRNAs already compacted, and usually in the presence of an excess of cationic liposomes when the ratio $m_{L}{/m}_{\mathrm{siRNA}}$ is high. A Boltzmann-type fit of the data in Figure 1a allowed us to determine the electroneutrality ratio of the lipoplexes at a $m_{L}{/m}_{\mathrm{siRNA}}=\;(5.7\pm 0.3)$. Using the protocol fully described previously [56,64], the electroneutrality ratio was used to determine the effective charge of C_3_(C_16_His)_2_, which was $q_{\mathrm{eff},\;\mathrm{GCL}}^{+}=\;(1.4\pm 0.1)$, a 30% lower than the nominal one (+2). This decrease has been attributed to the delocalization of the electric charge along the aromatic rings of the functionalized histidine residues, although other factors such as the packing density of the lipid chains have been reported to influence on the protonation state of the lipid [65]. This effect has also been observed in lipoplexes formed by the lipid mixture C_3_(C_16_His)_2_/DOPE and pDNA [26] and in others lipoplexes that contain GCL lipids bearing the imidazolium ring in their structure [56,66]. By contrast, the effective charge of siRNA is considered to be the same as its nominal one ($q_{\mathrm{eff},\;\mathrm{siRNA}}^{-}=\;-2/\mathrm{bp}$), as reported in the literature for either linear DNA (with thousands of base pairs, as calf thymus or salmon sperm DNA) [56,57,58] or short RNAs (with 19–25 bp) [38,67]. Considering these results, it can be concluded that the formation of lipoplexes is mainly driven by electrostatic forces between the oppositely charged cationic lipid and the anionic siRNA molecules. There is also an important entropic factor associated with the release of Na^+^ counterions to the bulk solution when the lipoplex is formed [58,67,68]. The values of the effective charges allow us to work with effective charge ratios (ρ_eff_) in the subsequent experiments:

(2) $$\rho_{\mathrm{eff}}=\frac{n^{+}}{n^{-}}=\frac{q_{\mathrm{eff},\;\mathrm{GCL}}^{+}{(m}_{\mathrm{GCL}}{/M}_{\mathrm{GCL}})}{q_{\mathrm{eff},\;\mathrm{siRNA}}^{-}{(m}_{\mathrm{siRNA}}{/M}_{\mathrm{siRNA}})}$$

where $n^{+}$, $n^{-}$, $q_{\mathrm{eff},\;\mathrm{GCL}}^{+}$, $q_{\mathrm{eff},\;\mathrm{siRNA}}^{-}$, $M_{\mathrm{GCL}}$, and $M_{\mathrm{siRNA}}$ are the number of moles of positive (GCL) and negative (siRNA) charges, effective charges of GCL and siRNA per bp, and the molecular weight of GCL and siRNA per bp, respectively.

With regard to the efficiency of C_3_(C_16_His)_2_/MOG mixed lipids to compact the nucleic acid, Figure 1b reports the results of the agarose gel electrophoresis experiment performed. In this experiment, the GelRed probe, which is present in the agarose gel, gets intercalated within the hydrophobic environment that represents the double-stranded helix of unprotected siRNA, increasing the probe quantum emission yield and, in turn, the intensity of its fluorescence emission. Thus, the characteristic fluorescent band seen in the first lane of the agarose gel, where the free siRNA was loaded as a control, helps to identify the uncompacted siRNA. Another fluorescent band is also visible in the second lane where the mass ratio $m_{L}{/m}_{\mathrm{siRNA}}$ is 2.7 (ρ_eff_ = 0.5). At higher lipid mixture contents (third and fourth lanes), the siRNA fluorescence band disappears, confirming the total compaction of siRNA by the C_3_(C_16_His)_2_/MOG lipid mixture once the electroneutrality ratio is overcome (ρ_eff_ > 1), which is in remarkably good agreement with ζ results.

### 3.2. Structural Study. Cryo-TEM and SAXS

**Figure 2:**
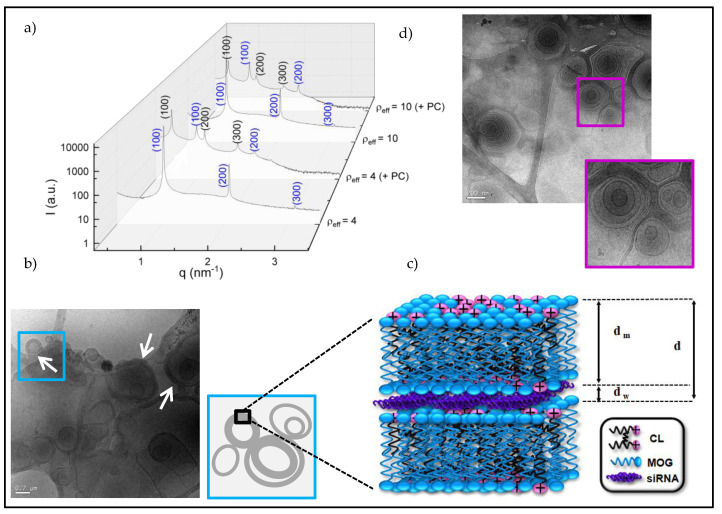
(**a**) SAXS diffractograms of the C_3_ (C_16_His)_2_/MOG-siRNA lipoplexes at α = 0.2 and ρ_eff_ = 4 and 10 in the absence and presence of human serum (+ PC). (**b**) Cryo-TEM micrograph of the lipoplexes at ρ_eff_ = 10 and α = 0.2. (**c**) Scheme included the multilamellar structure of lipoplexes aggregated in cluster-type shapes. (**d**) Cryo-TEM micrograph of C_3_(C_16_His)_2_/MOG lipid mixture. Scale bars of micrographs at 200 nm.

Once the C_3_(C_16_His)_2_/MOG mixture was confirmed to compact adequately siRNA molecules, it was convenient to gain insight into the structure and aggregation pattern of the lipoplexes, another factor of critical importance for a successful transport, cell uptake, and delivery of the cargo materials inside the cells. In this regard, SAXS and cryo-TEM techniques have already revealed their power, mostly when they are used together. SAXS diffractograms (plots of intensities vs. the momentum transfer vector, q) were obtained for C_3_(C_16_His)_2_/MOG-siRNA lipoplexes at ρ_eff_ = 4 and 10, and are collected in Figure 2a. These ρ_eff_ values were chosen to assure a good compaction level of the siRNA (ρ_eff_ > 1, see Figure 1b) and also because they were tested with successful results in other nanocarriers of pDNA or siRNA previously reported by us [26,43]. As can be observed in Figure 2a, the Bragg peaks can be correlated with the Miller indexes of a lamellar L_α_ lyotropic liquid crystal phase ((hkl) = (100), (200) and (300)) in both conditions (blue labels in Figure 2a). This multilamellar arrangement can be interpreted as a sandwich-type structure with alternating bilayers of C_3_(C_16_His)_2_/MOG mixed lipid and an aqueous layer between each pair of lipid bilayers, where the siRNA molecules with their counterions are located. Cryo-TEM experiments, also run for the lipoplexes at ρ_eff_ = 10, confirmed the multilamellar arrangement found in SAXS diffractograms, as shown in the micrograph reported in Figure 2b as an example. One can observe in this micrograph an appreciable population of nanostructures with the walls clearly thickened and deformed (see arrows in the figure), revealing how the presence of siRNA induces liposome aggregation to form cluster-type lipoplexes. Figure 2c shows a chart of a zoom view of this aggregation pattern and a scheme of the overall lamellar L_α_ phase. The same multilamellar pattern was found in a previous study where C_3_(C_16_His)_2_ was combined with DOPE to transfect pDNA [26]. However, this contrasts with the bicontinuous lyotropic liquid crystal cubic phases found by us for lipoplexes constituted by siRNA and a lipid mixture of an arginine-based cationic lipid and MOG [43]. Nanotube- or ribbon-type structures, found in some lipoplexes constituted by pDNAs and a lipid-based nanovector with amino acid residues in its structure [17,53] associated with low or zero levels of transfection, were not observed herein. As indicated in the scheme included in Figure 2c, the interlamellar distance (d) of the L_α_ phase (also known as the periodicity of the structure) can be expressed as the sum of the thicknesses of the lipid bilayer (d_m_) and the aqueous layer (d_w_). SAXS experiments allowed us to determine d from the q factors at which the Bragg peaks are found in the diffractograms (${d=\;2\pi n\;/\;q}_{\mathrm{hkl}}$, where n is the scattering order), as summarized in Table 1. As seen in this table, the interlamellar distance is not affected by the lipoplex composition (ρ_eff_). Thus, an average value of (6.1 ± 0.3) nm has been obtained, in agreement with those found for other multilamellar lipoplexes previously reported [18,26]. On the other hand, d_m_ was estimated around 4.5 nm from cryo-TEM micrographs where liposomes were found (see Figure 2d, as an example) and also with Tanford’s model [69,70,71]. Thus, d_w_ was calculated (= d − d_m_) around 1.6 nm, a thickness suitable for the accommodation of siRNA in between cationic lipid bilayers [38,72].

SAXS experiments were also carried out in the presence of HS (indicated with +PC in the diffractograms), with the aim of studying how the protein corona that surrounds the lipoplexes in a biological medium affects their structures. It is noticeable that the presence of proteins did not modify the original L_α_ lamellar structure (shown also in blue in Figure 2a), but favored the formation of an additional L_α_ lamellar phase at lower q values (shown in black in Figure 2a). Table 1 also reports the values of the interlamellar distance for these two structures. As can be noticed, d is not affected by the presence of proteins in the original L_α_ phase (d = 6.2 ± 0.3) nm), while it is longer for the secondary L_α_ structure (d = (10.8 ± 0.5) nm), corroborating that the proteins are most likely surrounding the lipoplex surface. It has been already reported in the literature that the incubation of lipoplexes in plasma or serum produces extra peaks in the SAXS diffractograms of pDNA-lipoplexes [73] and siRNA-lipoplexes [43], which were also explained in terms of the coexistence of more than one lyotropic liquid crystal phases, with different levels of compaction.

### 3.3. In Vitro Studies

**Figure 3:**
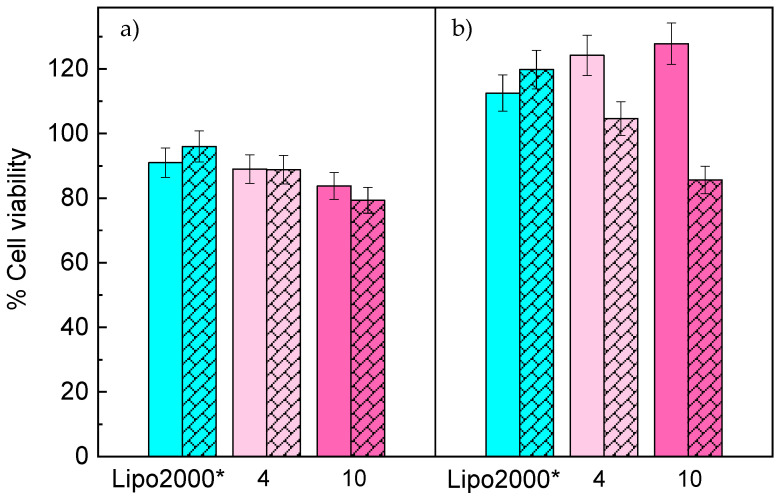
Percentage of cell viability of HeLa-GFP (**a**) and T731-GFP (**b**) cell lines in the presence of C_3_(C_16_His)_2_/MOG-siRNA lipoplexes (pink bars) at α = 0.2 and ρ_eff_ = 4 and 10. The % of cell viability was normalized to those obtained for untreated cells. Unstriped and striped bars correspond to 48 and 72 h of incubation, respectively. The blue bar corresponds to commercial control Lipo2000*-antiGFP-siRNA, used here as positive control. The experiments were performed with 10% (*v/v*) of HS, the data represent the mean of three wells, and errors are within ±5%.

To consider whether the formed lipoplexes can be useful as nanovectors for siRNA delivery into cells and their use in prospective in vivo applications, they must fulfill a series of conditions such as biocompatibility with cells and tissues, and controlled release of their cargo at the site of action, among others. In order to test the potential cytotoxicity of the present lipid-based nanocarriers, a preliminary evaluation of the in vitro cell viability of two different cancer cell lines in the presence of C_3_(C_16_His)_2_/MOG-siRNA lipoplexes at ρ_eff_ = 4 and 10 was done by means of the CCK-8 proliferation assay. Figure 3 shows the % of cell viability of HeLa-GFP (a) and T731-GFP (b) cells after 48 (unstriped bars) and 72 h (striped bars) of incubation in the presence of the different formed lipoplexes. Non-treated cells were considered as the negative control (100% of cell viability), and cells treated with the commercial transfecting reagent Lipo2000* were considered as the positive ones. 

Viabilities above 80% for both types of lipoplexes (pink bars) were found in both cell lines, corroborating the excellent biocompatibility of the present lipid-based nanocarriers. This small cytotoxicity agrees with that typically found for other lipid nanostructures containing histidine and other amino acid-based moieties [18,26,43,74,75,76], an advantage that prompts the consideration of using these nanovectors in future in vivo applications. It is important to notice that viabilities of T731-GFP cells were slightly larger than those obtained for HeLa-GFP ones, in agreement with previous works [43]. This can be a consequence of the faster cell division cycle of T731 cells and the phenotype differences between both types of cell lines herein used [77,78].

**Figure 4:**
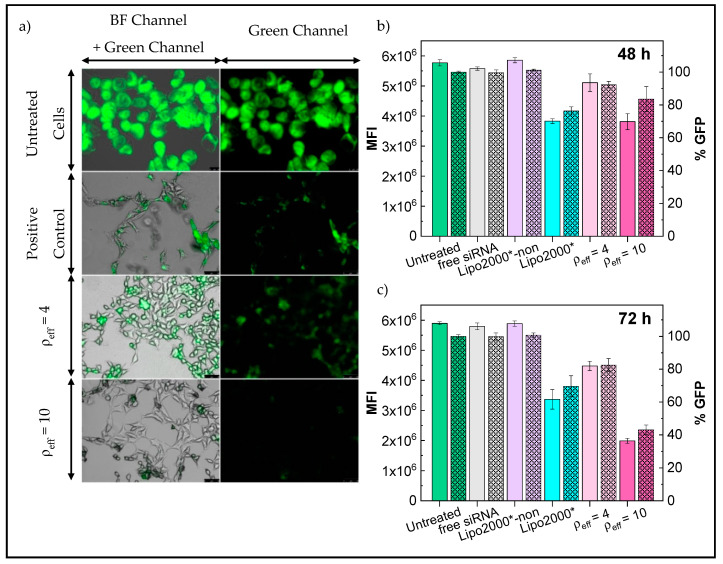
(**a**) Epifluorescence microscopy images: Merged images (bright field (BF) + green fluorescence channel (left)) and green fluorescence channel (right) for HeLa-GFP cells treated with C_3_(C_16_His)_2_/MOG-siRNA lipoplexes (α = 0.2; ρ_eff_ = 4 and 10) and Lipo2000*-antiGFP-siRNA as positive control, after 72 h of incubation. Scale bars are 50 μm, except untreated cells which are 10 μm. GFP knockdown levels expressed as MFI (unstriped bars) and % GFP (striped bars) for HeLa-GFP cells at 48 (**b**) and 72 h (**c**) of incubation. Cells treated by the lipoplexes were collected at α = 0.2 and ρ_eff_ = 4 (light pink bars) and 10 (dark pink bars). Untreated cells were used as a reference of the highest MFI and % GFP value. Free siRNA and Lipo2000*-non-targeting siRNA were used as negative controls (gray and purple bars, respectively) while Lipo2000*-antiGFP-siRNA was used as positive control (blue bars). All experiments were performed with 10% (*v/v*) of HS. The data represent the mean ± SD of three independent wells.

Additionally, with the aim of assessing the potential gene knockdown activity of these cell-friendly gene nanocarriers, epifluorescence microscopy was used to monitor the fluorescence signal changes from overexpressed GFP in both cancer cell lines (HeLa-GFP and T731-GFP) upon 72 h of incubation of the lipoplexes in HS-supplemented culture medium, which favors the PC formation around lipoplexes. For HeLa-GFP cells (Figure 4a), green channel and merged (green and bright field channels) images were used to locate and analyze the GFP expression. From these images, no significant differences in the GFP fluorescence levels between the positive control Lipo2000*-antiGFP-siRNA (row 2) and the lipoplexes formed at ρ_eff_ = 4 (row 3) were detected; moreover, relevant decreases in GFP fluorescence were observed (higher knockdown activity) for those lipoplexes formed at ρ_eff_ = 10 (row 4). ImageJ software was used to make a semi-quantitative estimation of the silencing activity of the lipid-based nanocarriers by measuring the remaining GFP fluorescence intensity after treatment. In this regard, values of 57, 68 and 12% of the initial fluorescence shown by non-treated cells were obtained for the Lipo2000*-antiGFP-siRNA control and lipoplexes formed at ρ_eff_ = 4 and 10, respectively. Similar % GFP values were found in additional images analyzed (Figure S1).

Flow cytometry analysis was carried out to make a quantitative analysis that confirms the trends observed with epifluorescence images. Figure 4 shows MFI (unstriped bars) and % GFP (striped bars) values of HeLa-GFP cells obtained at 48 (Figure 4b) and 72 h (Figure 4c) post-incubation with Lipo2000*-antiGFP-siRNA (positive control, blue bars) and C_3_(C_16_His)_2_/MOG-antiGFP-siRNA lipoplexes (at ρ_eff_ = 4, light pink bars, and ρ_eff_ = 10, dark pink bars). Free added antiGFP-siRNA (gray bars) and a non-targeting siRNA sequence compiled to Lipo2000* (Lipo2000*-non, purple bars) were used as additional controls. From Figure 4, it is clear that there was need for a vector-assisted delivery strategy to protect siRNA from nuclease degradation in the biological environment in order to exert its knockdown activity, as confirmed by the absence of any sign of decrease in MFI and % GFP values in these GFP-overexpressing HeLa cells (untreated cells, green bars) after administration of free antiGFP-siRNA. Also, the reduction of the MFI (or % GFP value) was a consequence of the antiGFP-siRNA sequence, since the cells treated by the non-targeting siRNA showed the same values of MFI or % GFP as the untreated ones. It is also remarkable in Figure 4 that there is a relatively noticeable knockdown activity of C_3_(C_16_His)_2_/MOG-siRNA lipoplexes formed at ρ_eff_ = 10 after 48 h of incubation, with decreases of ca. 33 and 20% in MFI and % GFP, respectively (Figure 4b), which are rather similar to those obtained with the standard Lipo2000* control. Conversely, the inhibitory effect of lipoplexes formed at ρ_eff_ = 4 was less intense, with reductions around 8–12% for both quantities. At longer incubation times (72 h), the gene silencing effect was much more intense (Figure 4c). Silencing activities of ca. 25 and 17% in MFI and % GFP, respectively, were detected for C_3_ (C_16_His)_2_/MOG-siRNA lipoplexes formed at ρ_eff_ = 4, while these values increase to 66 and 57% at ρ_eff_ = 10. For the latter, the achieved knockdown activity is almost two-fold larger compared to that of the Lipo2000* positive control.

**Figure 5:**
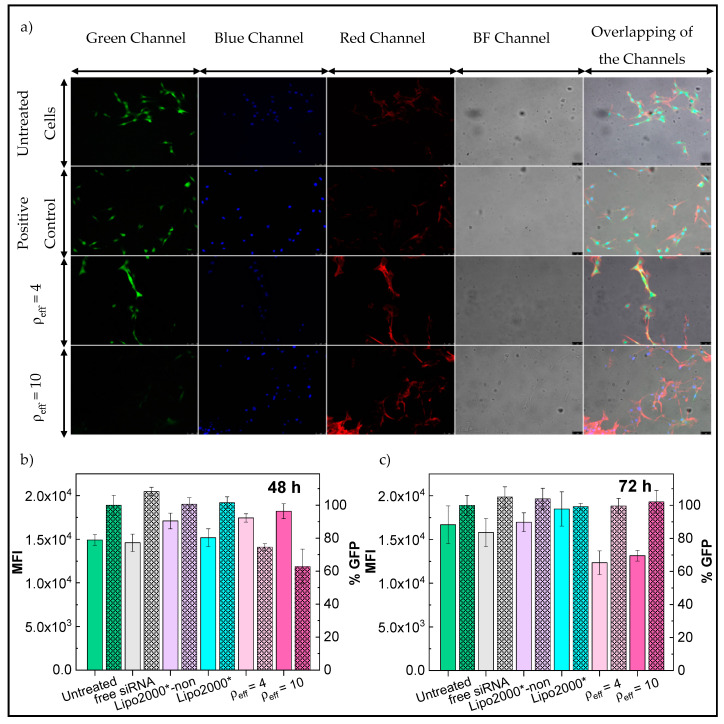
(**a**) Epifluorescence microscopy images for T731-GFP cells treated with C_3_(C_16_His)_2_/MOG-siRNA lipoplexes (α = 0.2; ρ_eff_ = 4 and 10) and Lipo2000*-antiGFP-siRNA as control, after 72 h of treatment. Images were collected in different channels of fluorescence signals and the overlapping of them. Scale bars are 75 μm. GFP knockdown levels expressed as MFI (unstriped bars, left y-axis) and % GFP (striped bars, right y-axis) in T731-GFP cells at 48 (**b**) and 72 h (**c**) of incubation. Cells treated by the lipoplexes were collected at α = 0.2 and ρ_eff_ = 4 (light pink bars) and 10 (dark pink bars). Untreated cells were used as a reference of the highest MFI and % GFP value. Free siRNA and Lipo2000*-non-targeting siRNA were used as negative controls (gray and purple bars, respectively) while Lipo2000*-antiGFP-siRNA was used as positive control (blue bars). All experiments were performed with 10% (*v/v*) of HS. The data represent the mean ± SD of three independent wells.

On the other hand, the gene knockdown activity of the developed lipid-based nanocarriers was additionally tested in the T731-GFP mice astrocyte cell line (Figure 5). Firstly, % GFP values were obtained from epifluorescence microscopy images using ImageJ to have a better definition of the protein signal distribution inside the cells, as mentioned previously. In this case, different channels were used to define the signals from several cell components, that is, the GFP signal appeared in the green channel (Figure 5a, first column), cell nuclei in the blue one (Figure 5a, second column), the actin filaments of the cell cytoplasm in the red one (Figure 5a, third column), and the complete cell morphology in bright field (Figure 5a, forth column). Calculated % GFP values were 87, 69 and 40% for cells treated with Lipo2000*-antiGFP-siRNA and C_3_(C_16_His)_2_/MOG-siRNA lipoplexes at ρ_eff_ = 4 and 10, respectively. Similar percentages were obtained in additional analyzed images (Figure S2). These results corroborate the observed clear existence of an effective knockdown activity of C_3_(C_16_His)_2_/MOG-siRNA lipoplexes, especially when compared to the commercial Lipo2000* transfecting reagent.

According to flow cytometry analysis, and in contrast to the observations with HeLa-GFP cells, no apparent decreases in GFP fluorescence (neither in MFI or % GFP) were observed for Lipo2000*-antiGFP-siRNA when compared to untreated T731-GFP cells after 48 h of incubation. Conversely, the % GFP signal was largely reduced for C_3_(C_16_His)_2_/MOG-siRNA lipoplexes at ρ_eff_ = 4 and 10 in ca. 25 and 37%, respectively (Figure 5b), thanks to the silencing activity of siRNA in an important number of cells, although those still fluorescently active have large amounts of overexpressed GFP. However, at longer incubation times (Figure 5c), the opposite trend was noted: MFI signals decreased in the presence of C_3_ (C_16_His)_2_/MOG-siRNA lipoplexes (at both conditions ρ_eff_ = 4 and 10) by ca. 24%, but not for the Lipo2000*-antiGFP-siRNA control, whereas % GFP kept constant at ca. 99% in all cases. This trend may arise from a balance between the well-sustained knockdown activity of the siRNA complexed in the nanovectors and the faster division cycle of T731-GFP cells, which favors a progressive increment in the number of fluorescently-active cells bearing a lower intensity, a consequence of the splitting of the active siRNA between the dividing cells. This behavior, already observed in previous works using this cell line [43] clearly reveals the role played by cell phenotype in cell viability (see Figure 3b), nanovector uptake and internalization, and subsequent therapeutic efficiency [79].

In conclusion, considering all the in vitro results above presented, it can be remarked the noticeable gene knockdown capacity of the C_3_(C_16_His)_2_/MOG-siRNA lipoplexes herein studied, particularly at ρ_eff_ = 10 in both cancer cell lines, is in agreement with what we have recently published for an arginine-based siRNA nanocarrier [43]. Therefore, it seems there is a correlation between larger GFP expression inhibition and higher lipid content in the siRNA nanocarrier, without this implying an enhancement in its cytotoxicity, as herein demonstrated. Furthermore, it is worth noting that the C_3_(C_16_His)_2_ gemini cationic lipid used in this work mixed with MOG also demonstrated high efficiency in transfecting a pDNA, which in that case mixed with DOPE [26]. These two evidences support the GCLs or CLs functionalized with histidine-based head groups a compelling and striking versatility in the gene therapy field. Certainly, the delocalized cationic charge in both head groups of the GCL can promote strong electrostatic interactions with the anionic nucleic acids, enabling effective compaction and protection of the cargo by the lipid-based nanocarrier. Undoubtedly, the fusogenic properties of either MOG or DOPE (the helper lipids) are also key to facilitating the endocytic entry of the genetic material into the cell. However, the present satisfactory results obtained in vitro might not necessarily translate to an in vivo environment since its biological complexity can affect the capabilities and properties of the nanocarrier. In fact, many nanosystems with promising in vitro outcomes, then fail in in vivo tests because the conditions of the biomimicked physiological environment are different from those of the real physiological environment, being the high presence of proteins one of its most outstanding peculiarities. For this reason, the last part of this study will be focused on the identification of the proteins with higher affinity for the surface of C_3_(C_16_His)_2_/MOG-siRNA lipoplexes.

### 3.4. Protein Corona Characterization

**Figure 6:**
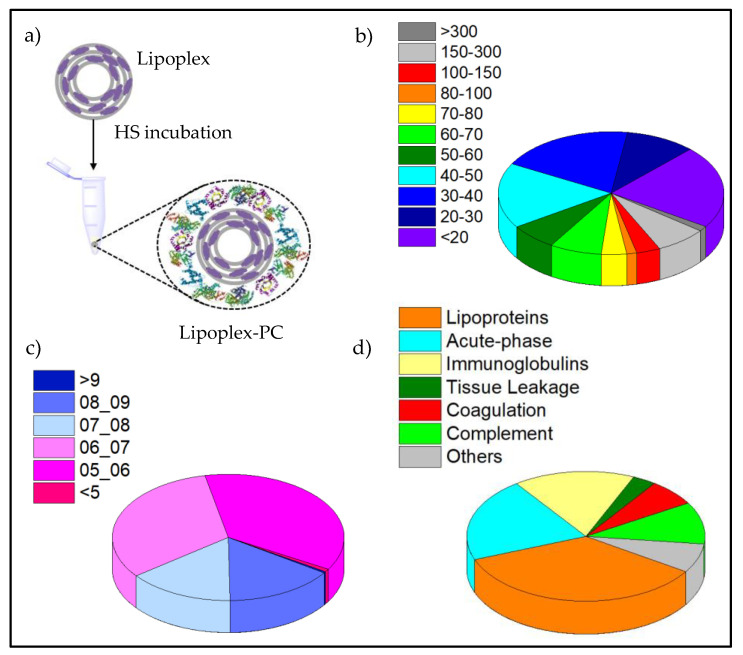
(**a**) Scheme depicting protein corona formation (PC). Percentage of the proteins found within the protein corona (PC) of C_3_(C_16_His)_2_/MOG-siRNA lipoplexes at α = 0.2 and ρ_eff_ = 10, classified by their molecular weight, MW in kDa (pie chart **b**), by their isoelectrical point, pI (pie chart **c**) and by their physiological function (pie chart **d**).

As already commented, once the lipoplexes are in contact with human plasma, the proteins attached to the surface will strongly influence their biological behavior. After all, what cells firstly see is not the lipoplex surface but the PC adsorbed on it (see scheme on Figure 6a). As a result, it becomes of utmost importance the analysis of the protein corona (PC) to determine its nature and composition. The C_3_(C_16_His)_2_/MOG-siRNA lipoplexes were thus analyzed by nanoLC-MS/MS at the most promising gene knockdown condition found in vitro, ρ_eff_ = 10, after 1 h of incubation with HS. The relative percentages of the distinct proteins were determined and, in turn, proteins were classified according to their molecular weight (MW), isoelectric point (pI), and physiological function, as shown in Figure 6b, c and d, respectively (see also Table S1). Figure 6b indicates that proteins with low molecular weight (< 50 KDa) were preferentially adsorbed onto the nanocarrier surface (~70%), and only ~9% of the total amount of adsorbed proteins presented molecular weights above 150 kDa. On the other hand, C_3_ (C_16_His)_2_/MOG-siRNA lipoplexes exhibited strong affinities by proteins with a pI below 7, as can be inferred in Figure 6c. The fact that about 70% of adsorbed proteins showed negative charge at physiological pH is in agreement with the hypothesis that the PC formation is mostly driven by electrostatic interaction between negatively charged proteins and cationic lipids, as observed in previous PC studies [43,50].

Finally, the classification made according to the physiological function revealed that the PC formed around the lipoplexes is mainly comprised of lipoproteins, acute phase, and immunoglobulins proteins (35%, 21%, and 16% respectively). In contrast, tissue leakage, coagulation, and complement proteins were found in a considerable minor proportion (3%, 7%, and 10%, respectively). Lipoproteins are involved in intracellular trafficking and can promote longer bloodstream circulation times, while immunoglobulins and acute phase proteins are related to immune and inflammatory responses. The complement proteins usually favor the elimination of nanocarriers from the systemic circulation, while coagulation proteins are involved in the coagulation process [80,81]. A further classification based on the percentage of total proteins identified for each functional class is displayed on Figures S3 and S4, while the top 25 most-abundant corona proteins are collected in Table 2. Within the lipoproteins, apolipoproteins A-I and A-II are the most abundant (13% and 7%, respectively), followed by serum albumin (5%). Owing to their capacity to prolong the circulation time in the bloodstream and their implication in the metabolism and transport of cholesterol, these proteins are recognized as dysopsonins. Although factors governing protein corona composition are still under discussion, the composition above described could help in explaining the notable knockdown silencing activity displayed by C_3_(C_16_His)_2_/MOG-siRNA lipoplexes. Thus, the combination of a moderate content of lipoproteins (ApoA-I and ApoA-II in particular) and a low content of complement proteins could be favoring a longer circulation of the present lipid-based nanocarriers in the bloodstream and a lower interaction with phagocytic cells [82], which could justify, at least in part, the good silencing outcomes herein obtained; nevertheless, more specific studies in this regard would be necessary.

Additionally, it is remarkable the similarity between the global composition of the PC of C_3_(C_16_His)_2_/MOG-siRNA lipoplexes studied in this work (reported in Figure 6) and that found in a previous one reported by our group for an arginine-based lipid vector that, mixed with MOG and siRNA, yielded C_12_ANHC_18_/MOG-siRNA lipoplexes [43]. In both cases, the cationic lipid (CL or GCL) incorporated a functionalized amino acid residue in its structure, either arginine or histidine type. In both cases, moderate or even high silencing efficiencies were found in the same cell lines (HeLa-GFP and T731-GFP). What differs in these two systems is the structure that the lipid mixture adopted when the siRNA was compacted to form the lipoplexes. In the case of C_12_ANHC_18_/MOG-siRNA lipoplexes, two coexisting bicontinuos lyotropic liquid crystal cubic phases were found in SAXS experiments [43], while in the present case, SAXS results have demonstrated that C_3_(C_16_His)_2_/MOG-siRNA lipoplexes arranged in two coexisting L_α_ lamellar phases. Taking all these evidences into account, it could be affirmed that, even assuming that the lipoplex structure could have an important weight in the efficiency of the transfection and/or silencing process, as it has been admitted until now, the nature of the proteins adsorbed on the surface lipoplex (i.e., PC composition) might have a critical influence in their biological response, as demonstrated in previous works [83,84]. In addition, it seems that the introduction of amino acid residues on the cationic head of the lipid-based nanovector somehow conditions the adsorption of those proteins that tend to favor the permanence of the gene nanocarrier in the bloodstream, thus promoting the transfection/silencing process, although more specific assays would be necessary to affirm it unambiguously.

## 4. Conclusions

The gemini cationic lipid with functionalized histidine groups in its structure, C_3_(C_16_His)_2_, in combination with the neutral helper lipid MOG (α = 0.2), has demonstrated in this work its potential to compact, protect and transfect siRNA in two GFP over-expressing cancer cell lines (HeLa-GFP and T731-GFP), provoking in turn the GFP knockdown with efficiency and cell-safety. This affirmation is based on the biophysical study herein presented that has combined both physicochemical and biochemical experiments to understand the interactions between siRNA and the lipids; the structural patterns of the resulting lipoplexes; and their capacity to cross the cellular membrane, deliver the nucleic acid in the cellular cytoplasm, and knockdown the GFP expression. The histidine-based GCL provided the necessary positive and delocalized charge to compact the anionic siRNA molecules by means of a strong electrostatic interaction with an important entropic component associated with the release of Na^+^ counterions to the bulk. It was also demonstrated that C_3_(C_16_His)_2_/MOG-siRNA lipoplexes were arranged in a L_α_ lamellar lyotropic liquid crystal phase, in coexistence with an additional L_α_ phase at lower q values when the lipoplexes were incubated with HS, this second lamellar phase being compatible with a less compacted structure where the proteins of HS were surface-adsorbed. This coexistence of phases could favor once again the uptake of the lipoplex by the cell, revealing a structure-activity relationship in the GFP knockdown evidences found. On the other hand, C_3_(C_16_His)_2_/MOG-siRNA lipoplexes displayed an excellent biocompatibility in both HeLa-GFP and T731-GFP cells. Moreover, they efficiently induced GFP knockdown in both cell lines, with even better outcomes than those shown by the standard Lipo2000*-siRNA, ρ_eff_ = 10 being the most promising lipoplex composition. The PC surrounding the lipoplex surface was confirmed to be mainly composed by proteins with pI less than 7 (as expected for cationic nanoaggregates) and with MW lower than 50 kDa. Furthermore, it was found that the most abundant proteins were apolipoproteins (A-I and A-II) and serum albumin, while complement proteins were found in low percentages. This particular combination may help in explaining the remarkable knockdown activity of C_3_(C_16_His)_2_/MOG-siRNA lipoplexes in terms of a longer circulation time of the nanocarrier in the bloodstream and a lower interaction with phagocytic cells, although more specific studies would be required in this respect. Finally, comparing the results herein obtained with previous studies (above referenced), it can be also concluded that the GCL C_3_(C_16_His)_2_, with histidine-based polar heads, can be recommended as a versatile and promising option in both gene transfection (pDNA) and gene silencing (siRNA) strategies.

## Figures and Tables

**Table 1:** Values of the periodic distance of the lamellar structure, d, for C_3_(C_16_His)_2_/MOG-siRNA lipoplexes at α = 0.2 and ρ_eff_ = 4 and 10 in the absence and presence of human serum (+ PC). The primary L_α_ structure is in blue, and the secondary L_α_ structure is in black, the same color codes as in diffractograms (Figure 2). Values of q_hkl_ (nm ^−1^) and d_hkl_ (nm) are estimated with a 5% error.

α = 0.2	C_3_ (C_16_His)_2_/MOG-siRNA			
q_hkl_ or d_hkl_	ρ_eff_ = 4	ρ_eff_ = 4 (+ PC)	ρ_eff_ = 10	ρ_eff_ = 10 (+ PC)
q_100_	-	0.6	-	0.6
	1.0	1.0	1.0	1.0
d_100_	-	10.6	-	10.8
	6.0	6.2	6.0	6.2
q_200_	-	1.1	-	1.2
	2.1	2.0	2.1	2.0
d_200_	-	10.9	-	10.8
	6.1	6.2	6.0	6.2
q_300_	-	1.7	-	1.7
	3.1	-	3.1	-
d_300_	-	10.9	-	10.8
	6.1	-	6.1	-

**Table 2:** Top 25 most-abundant proteins present in the protein corona surrounding C_3_(C_16_His)_2_/MOG-siRNA lipoplexes surfaces at α = 0.2 and ρ_eff_ = 10.

Protein Number	Description	%
1	Apolipoprotein A-I	12.85
2	Apolipoprotein A-II	7.39
3	Serum albumin	5.38
4	Ig kappa constant	3.73
5	Ig lambda constant 2	3.13
6	Alpha-1-antitrypsin SV = 3	2.97
7	Alpha-1-antitrypsin SV = 1	2.94
8	Apolipoprotein A-IV	2.90
9	Complement C3	2.89
10	Apolipoprotein E	1.99
11	Apolipoprotein C-III	1.81
12	Complement C4-B	1.72
13	Complement C4-A	1.67
14	Ig heavy constant mu	1.62
15	Trypsin	1.54
16	Apolipoprotein C-I	1.35
17	Retinol-binding protein 4	1.34
18	APOC4-APOC2 readthrough (NMD candidate)	1.31
19	Serotransferrin	1.25
20	Apolipoprotein B-100	1.22
21	SAA2-SAA4 readthrough	1.19
22	Haptoglobin	1.17
23	Vitronectin	1.10
24	Isoform 2 of Clusterin	1.09
25	Ig lambda-like polypeptide 5	1.05

## Supplementary Materials

The following are available online at https://www.mdpi.com/1999-4923/12/9/791/s1, Figure S1: Epifluorescence microscopy images: Merged images (bright field (BF) + green fluorescence channel (left)) and green fluorescence channel (right) for HeLa-GFP cells treated with C_3_(C_16_His)_2_/MOG-siRNA lipoplexes (α = 0.2; ρ_eff_ = 4 and 10) in the presence of 10% (*v/v*) of HS, after 72h of incubation, Figure S2: Epifluorescence microscopy images for T731-GFP cells treated with C_3_(C_16_His)_2_/MOG-siRNA lipoplexes (α = 0.2; ρ_eff_ = 4 and 10) in the presence of 10% (*v/v*) of HS, after 72h of incubation, Table S1: Relative percentage of proteins classified by their physiological function, by their MW in kDa and by their pI about pie charts in Figure 6, Figure S3: Classification of the most abundant proteins (lipoproteins, acute-phase and immunoglobulins proteins) found in the protein corona surrounding C_3_(C_16_His)_2_/MOG-siRNA lipoplexes surfaces, Figure S4: Classification of the proteins that constitute a minor fraction (tissue leakage, coagulation, complement and other proteins) present in the protein corona surrounding the C_3_(C_16_His)_2_/MOG-siRNA lipoplexes surfaces.
