# Supplementary material for: Protein Expression Knockdown in Cancer Cells Induced by a Gemini Cationic Lipid Nanovector with Histidine-Based Polar Heads
Source: Pharmaceutics. 2020 Aug 21;12(9):791. doi: 10.3390/pharmaceutics12090791 (PMC7558209; doi:10.3390/pharmaceutics12090791)
Supplement: Supplementary file 1 [file pharmaceutics-12-00791-s001.pdf]

pharmaceutics

Supplementary Materials: Protein Expression Knockdown in Cancer Cells Induced by a Gemini Cationic Lipid Nanovector with Histidine-Based Polar Heads

Natalia Sánchez-Arribas, María Martínez-Negro, Eva M. Villar, Lourdes Pérez, José Osío Barcina, Emilio Aicart, Pablo Taboada, Andrés Guerrero-Martínez and Elena Junquera

1. In vitro studies:

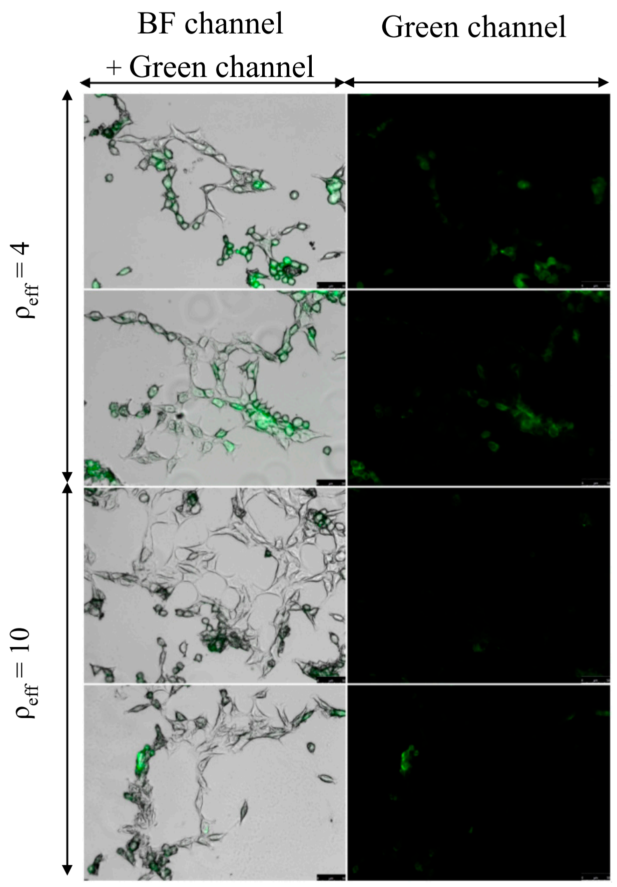

Figure S1. Epifluorescence microscopy images: Merged images (bright field (BF) + green fluorescence channel (left)) and green fluorescence channel (right) for HeLa-GFP cells treated with $C_3(C_{16}His)_2$/MOG-siRNA lipoplexes ($\alpha = 0.2$; $\rho_{eff} = 4$ and 10) in the presence of 10% (*v*/*v*) of HS, after 72 h of incubation. Scale bars are 50 μm. The % GFP values estimated by ImageJ were 52 and 48% at $\rho_{eff} = 4$ (row 1 and 2, respectively) and 14 and 16% at $\rho_{eff} = 10$ (row 3 and 4, respectively).

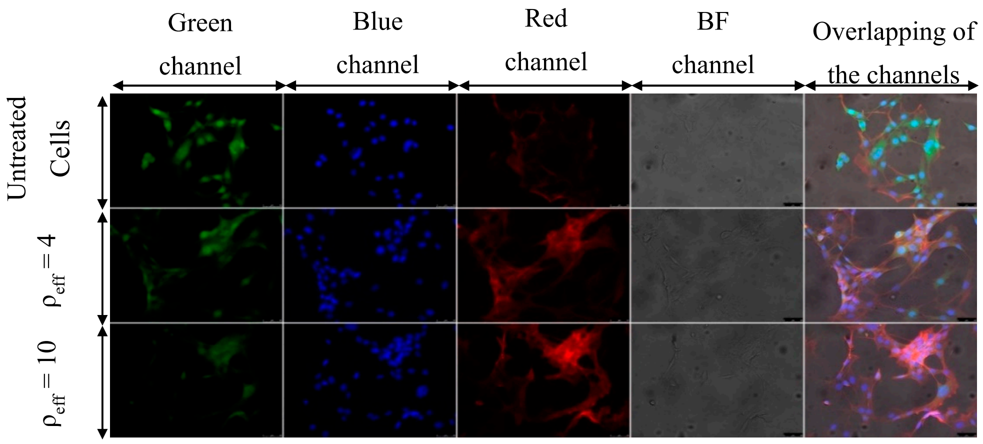

Figure S2. Epifluorescence microscopy images for T731-GFP cells treated with $C_3(C_{16}His)_2$/MOG-siRNA lipoplexes (α = 0.2; ρ_{eff} = 4 and 10) in the presence of 10% (*v*/*v*) of HS, after 72 h of incubation. Images were collected in different channels of fluorescence signals and the overlapping of them. Scale bars are 50 μm. The % GFP values estimated by ImageJ were 69% at ρ_{eff} = 4 (row 2) and 31% at ρ_{eff} = 10 (row 3).

2. Protein corona studies

Table S1. Relative percentage of proteins classified by their physiological function, by their MW in kDa and by their pI about pie charts in Figure 6.

Physiological Function	%
Lipoproteins	34.98
Acute-phase	20.95
Immunoglobulins	16.48
Tissue Leakage	3.17
Coagulation	6.67
Complement	10.47
Others	7.27

MW	%
>300	1.35
150-300	7.41
100-150	3.41
80-100	1.45
70-80	3.64
60-70	7.45
50-60	6.88
40-50	17.17
30-40	19.40
20-30	10.03
<20	21.81

pI	%
>9	0.32
08_09	15.26
07_08	14.84
06_07	32.18
05_06	36.63
<5	0.76

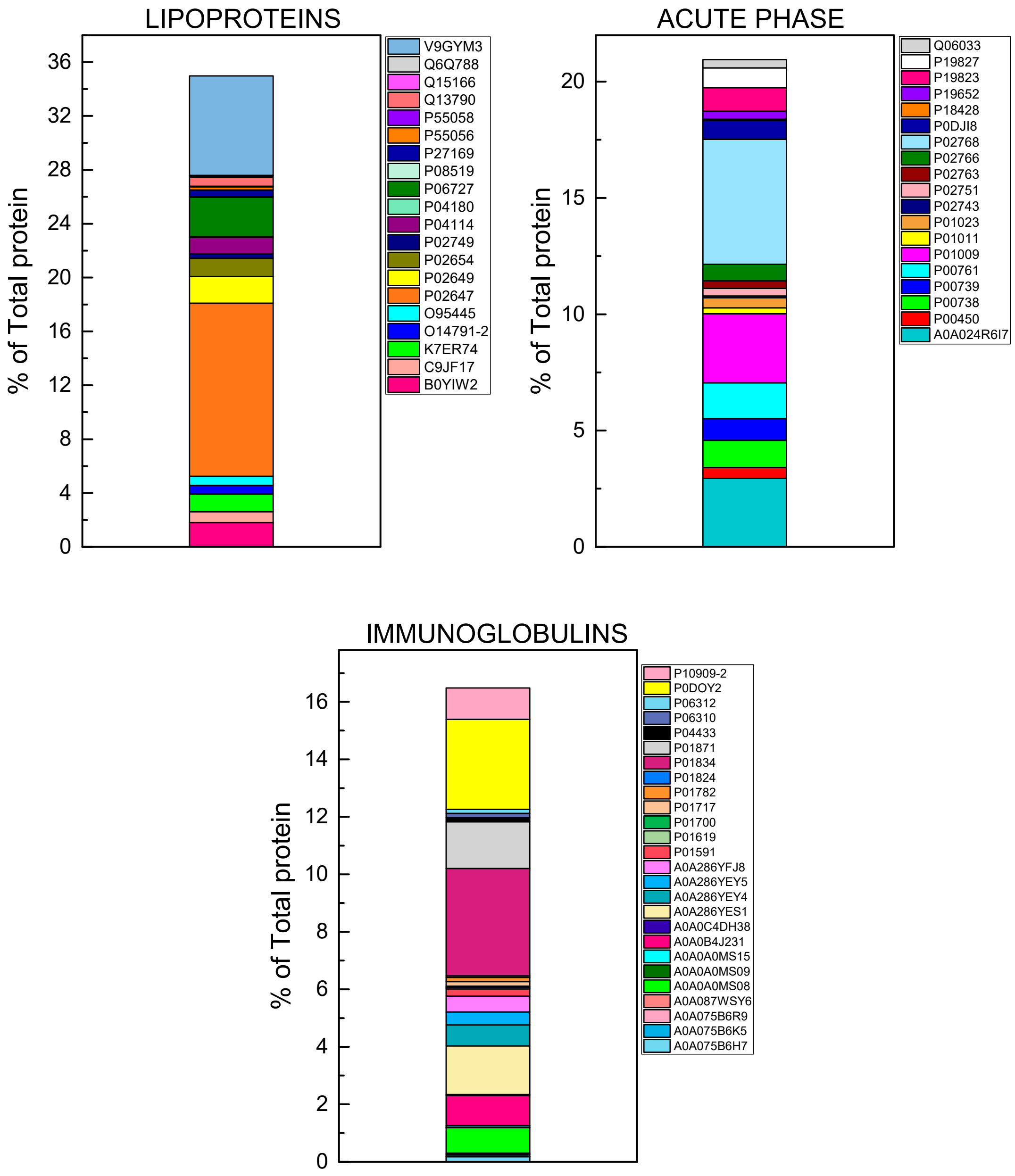

Figure S3. Classification of the most abundant proteins (lipoproteins, acute-phase and immunoglobulins proteins) found in the protein corona surrounding $C_3(C_{16}His)_2$/MOG-siRNA lipoplexes surfaces.

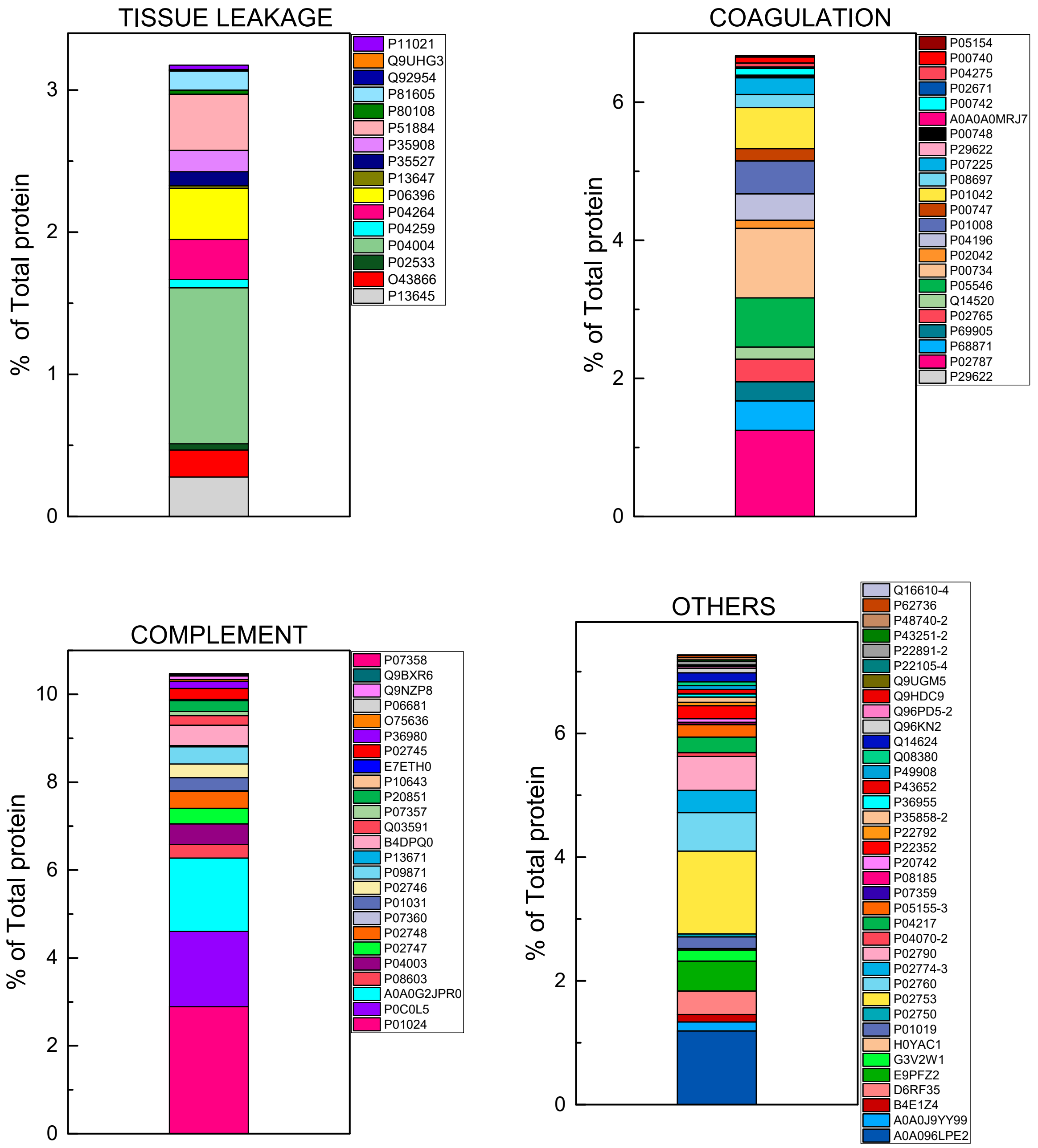

Figure S4. Classification of the proteins that constitute a minor fraction (tissue leakage, coagulation, complement and other proteins) present in the protein corona surrounding the $C_3(C_{16}His)_2$/MOG-siRNA lipoplexes surfaces.
